# “It Changes Your Orbit”: The Impact of Suicide and Traumatic Death on Adolescents as Experienced by Adolescents and Parents

**DOI:** 10.3390/ijerph17249356

**Published:** 2020-12-14

**Authors:** Karl Andriessen, Karolina Krysinska, Debra Rickwood, Jane Pirkis

**Affiliations:** 1 Centre for Mental Health, Melbourne School of Population and Global Health, The University of Melbourne, Parkville, VIC 3010, Australia; karolina.krysinska@unimelb.edu.au (K.K.); j.pirkis@unimelb.edu.au (J.P.); 2 Faculty of Health, University of Canberra, Canberra, ACT 2617, Australia; Debra.Rickwood@canberra.edu.au

**Keywords:** grief, bereavement, mental health, suicide, traumatic death, support, adolescents, family

## Abstract

Background: Having someone close die through suicide or another form of traumatic death is a distressing event in the lives of adolescents, putting them at risk of grief and mental health ramifications. As most research in this field has been focused on intrapersonal grief reactions, this study aimed to broaden the perspective by exploring the impact of the death through an interpersonal lens. Methods: The study involved individual and group interviews with bereaved adolescents (*n* = 20) and parents of bereaved adolescents (*n* = 18), and thematic analysis of the data. Results: The analysis yielded three themes: (i) the death is a life-changing experience, (ii) the death differentiates you from your peers, and (iii) the death impacts on the family system. Conclusions: The study revealed the devastating impact of the deaths on adolescents, their relationships with peers and the family system. Adolescents’ grief must be understood within the context of their agency and their immediate social environment. The findings clearly indicate that support for bereaved adolescents should incorporate the familial context.

## 1. Introduction

Experiencing the death of a close person, especially a parent, is considered to be the most disruptive and stressful event in the life of adolescents [1,2]. Approximately 4% of adolescents lose a parent, and about 78% lose a relative or a friend before the age of 18 [3,4,5]. Moreover, about one in 20 (4.6%) adolescents lose someone by suicide in one given year and about one in five (18%) do so at some point before they reach adulthood [6].

Common grief reactions in adolescents include crying and feelings of sadness, guilt and longing [7]. Although the grief processes after any type of death may be similar, some grief features may be more pronounced depending on the cause of death [8,9]. Adolescents bereaved by suicide may experience more feelings of shock, anxiety, anger, and self-blame than adolescents bereaved by natural causes. They may struggle more with “why” questions, and experience less social support [1,10]. Adolescents bereaved by suicide and other traumatic deaths have increased risk of depression, anxiety, post-traumatic stress disorder, and suicidal ideation in the first months after the bereavement, compared to those bereaved by natural deaths and compared to their non-bereaved peers [11,12]. Studies have also reported increased risk of depression in about 10% of adolescents bereaved by a suicide or other traumatic death of a parent more than two years after the loss [13,14]. 

Grief and mental health reactions after deaths with a sudden or traumatic nature, such as accidents and homicides, seem to resemble those of grief after suicide more than grief reactions after natural deaths both in adolescents and in adults [10,15,16,17].

The short and long-term impact of the loss in adolescents is affected by how the bereaved young person experiences and responds to the loss [18], and the young person’s perception of how the loss has changed their lives [4]. Our systematic review on suicide bereavement in adolescents revealed that pre-loss factors such as personal and family history of mental health, and post-loss factors such as the quality of the remaining relationships, affect the impact of the loss in adolescents [19].

To date, most bereavement research in adolescents, including suicide bereavement research, has focused on intrapersonal phenomena such as the bereaved adolescents’ grief experiences, coping styles and grief processes [7,19,20,21]. The few qualitative studies to-date have reported mostly on grief feelings, coping and personal growth experienced by bereaved adolescents (for example, Bartik et al. [22]; Silvén Hagström [23]).

However, people do not live or grieve in isolation [21,24], and as Cain (pp. 14–15) observed, grief processes after suicide “neither originate, nor evolve in an interpersonal vacuum” [25]. This is especially true for bereaved adolescents, who most often live in a family environment, and are faced with the key developmental tasks of building identity and autonomy [7]. Additionally, when deaths experienced by adolescents concern the loss of a nuclear family member, their grief reactions may interact with their immediate social environment. These observations point at the importance of involving the views and responses of parents in research as the bereaved children’s grief reactions are affected by the parents’ reactions to the child [18]. This study aimed to fill this gap as studies looking at the experiences of grief after suicide and other traumatic deaths in adolescents through an interpersonal lens are lacking.

This paper is part of a larger research project that examines what help should be provided to bereaved adolescents. Within that larger research project, we conducted a sizeable qualitative study involving individual and group interviews with bereaved adolescents, parents and clinicians (the APC study). The APC study examined participants’ experiences and recommendations on effective help for bereaved adolescents. We included parents as a target group as the literature indicated their potential role in facilitating adolescent help-seeking [26,27], and contributing to the effectiveness of help provided to adolescents bereaved by suicide or other traumatic deaths [28,29,30]. Involving parents also allowed us to study grief in adolescents through an interpersonal lens. Findings regarding what help should be provided to bereaved adolescents will be reported elsewhere. This paper aimed to investigate the question: what is the impact of suicide or other forms of traumatic death on adolescents as perceived by bereaved adolescents themselves as well as parents of bereaved adolescents?

## 2. Materials and Methods

### 2.1. Study Design and Sampling

We conducted the study according to the Consolidated Criteria for Reporting Qualitative Research [31]. We recruited a purposive sample of adolescents and parents located in Australia. Eligible adolescents had lost a family member or friend through suicide or other cause when the adolescent was aged between 12 and 18 years, and had experienced the loss between six months and 10 years before participating in the study. Parents could participate if they were the parent of an adolescent who met the above eligibility criteria. Adolescents and parents could participate irrespective of whether their parents or children participated.

We recruited participants from October 2019 to March 2020 through various bereavement and youth organizations, which disseminated the study announcement (for example, via their mailing lists, e-newsletter or social media). Participants were offered an AUD 30.00 supermarket voucher as reimbursement. Participants could choose whether to take part in a semi-structured individual telephone interview or a face-to-face group interview. Research has shown that individual interviews may be more effective in generating a broad range of issues, while sensitive and personal issues may be more often disclosed in a group discussion [32]. Offering multiple methods may also allow more potential participants to take part in qualitative research as their decision to participate is influenced by their perception of being able to contribute to the study via the given methods [33]. Based on the literature [34,35,36], and our experience in qualitative research [10,26,37], we estimated the sample size of adolescents and parents required to answer the research question to be between 15 and 20 participants each.

A total of 60 potential participants (31 adolescents, 29 parents) contacted us, and 20 adolescents and 18 parents participated in the study (Supplementary Table S1). Reasons for not participating were being ineligible (2 adolescents, 5 parents), unavailable (1 parent), emotional reasons (1 adolescent), and unknown reasons (8 adolescents and 5 parents). The adolescent participants (16 girls, 4 boys) were aged 14 to 26 years (M = 19.50, SD = 2.95). They had experienced the death on average 4 years previously (M = 3.92, SD = 2.49, range 1 to 10 years) when they were 12 to 18 years old. The deaths had occurred by suicide (*n* = 18) or by accident (*n* = 2), and most of the deceased persons were immediate family members: father (*n* = 9), brother (*n* = 2), sister (*n* = 2), mother (*n* = 2), other family (*n* = 2), and friend (*n* = 2). The parents (18 mothers) were aged 43 to 60 years (M = 53.20, SD = 4.35). The deaths had occurred on average 5 years ago (M = 5.31, SD = 2.89, range 1.5 to 10 years) when their adolescent child was 12 to 18 years old (M = 15.17, SD = 1.58). The deceased person was the child’s father (*n* = 10), brother (*n* = 4) or sister (*n* = 4), and the person had died by suicide (*n* = 13), accident (*n* = 2), manslaughter (*n* = 1), illness (*n* = 1) and undetermined (*n* = 1). The two deaths that were reported as “illness” and “undetermined” had also occurred in traumatic circumstances. As the analysis of the final interviews did not yield new data, we are sure that the sample size was adequate.

### 2.2. Procedure and Data Collection

We used a semi-structured interview guide, adapted for individual or group interviews, with open-ended questions allowing for probes and follow-up questions depending on how participants responded [38]. Parents were asked to report about their adolescent children, rather than on their own situation, although both situations were often intertwined. Interviews usually started with a question “Could you tell me about your/your adolescent’s bereavement experience?”. The next questions inquired about the needs that the adolescent/the adolescent child of the parent had experienced, how the needs had evolved over time, how the death had impacted on the adolescent’s life, their experiences with help-seeking and what they had found helpful. At the end of the interview participants had the opportunity to formulate advice for other bereaved adolescents. After the interview, participants received an evaluation form with five questions on how they had experienced taking part in the study (results will be reported separately).

Seven adolescents participated in an individual telephone interview and 13 others in 3 group interviews; 9 parents participated in a telephone interview, and 9 parents participated in 2 groups. The lead researcher (K.A.) conducted all interviews, except two, which were conducted by a second researcher (K.K.). Experienced counselors from the collaborating services co-facilitated the group interviews, which took place at their premises (see acknowledgments). Individual interviews with adolescents lasted on average 40 min (range 20–64 min), and group interviews took 70 min (range 53–80 min). The parents’ individual interviews lasted on average 53 min (range 29–76 min) and their group interviews 87 min (range 81–93 min). Interviews were audio-recorded, professionally transcribed and the researchers checked the de-identified transcripts for accuracy.

### 2.3. Research Team and Reflexivity

The study was based at the Centre for Mental Health in The University of Melbourne, which is a national and international leader in the field of mental health and suicide-related research. The lead researcher/interviewer was a social worker with ample experience in qualitative bereavement research with young people. The second interviewer was an experienced research psychologist and psychotherapist. The interviewers recorded field notes after the interviews, and during listening and reading the transcripts. No researcher had a prior relationship with the participants. The senior researchers and supervisors (D.R., J.P.) were renowned experts in the field of (youth) mental health, suicidal behaviour and help-seeking. The research team met regularly to minimize researcher bias and to ensure consistency throughout the study.

### 2.4. Data Analysis

We conducted a thematic analysis, which Braun and colleagues [39] labeled as “Codebook” Thematic Analysis. It consisted of an iterative process of systematically identifying and organizing patterns of meaning (i.e., themes) in a data set [40]. Two researchers (K.A., K.K.) conducted the analysis independently and followed the six iterative steps formulated by Braun and Clarke [40]. Analysis started with reading and rereading the data to produce initial codes. Next, we grouped the initial codes in potential themes, which involved the creation of mind maps (i.e., diagrams to visualize the data). Subsequently, we reviewed the codes and mind maps, and revised these against the data before deciding about the themes.

Initially, two researchers (K.A., K.K.) had created a codebook based on analysis of the same three transcripts [41]. The two researchers then coded the remaining transcripts. However, applying an inductive approach allowed the researchers to modify or create new codes, if needed, to capture the meaning of the data rather than its explicit content. We used NVivo 12 [42] for data management and settled any disagreement throughout the analysis by comparing notes and discussion.

### 2.5. Ethical Considerations

The Human Research Ethics Committee of The University of Melbourne approved the study (ID 1955213). All potential participants contacted us to express interest in the study. We strived to be as transparent as possible about the study and provided potential participants with the participant information, explained the purpose of the study, what participation involved, the voluntary basis of participation, and answered any questions. Participants provided written consent.

Based on the National Statement on Ethical Conduct in Human Research 2007 (Updated 2018) [43], and as with our previous studies [10,26], we used a different procedure for minors depending on their age. For minors aged 12–15 years, we obtained consent both from the adolescent and a parent/guardian. For minors aged 16–17 years, who potentially have the maturity to understand the research and consent, the researcher (K.A.) decided at the end of the initial contact whether a parent/guardian should consent. During the initial telephone contact, the researcher encouraged the adolescent to talk about the research participation with a parent/guardian, before or after the interview.

## 3. Results

The analysis resulted in three main themes: (i) the death is a life-changing experience, (ii) the death differentiates you from your peers, and (iii) the death impacts on the family system. Table 1 summarizes the themes. Participants’ names are fictional; “A” and “P” refer to an adolescent and parent participant, respectively.

### 3.1. Theme 1: The Death Is A Life-Changing Experience

Most young participants emphasized that experiencing the death was an alien experience that turned their life upside-down. From their perspective, the deaths had occurred suddenly and unexpectedly. Even if there had been, for example, prior suicide attempts, they did not feel prepared for the death occurring. Some of the young participants had found the deceased person, which reinforced the traumatic nature of the death. All young participants and parents underlined the immediate emotional impact of the death. Common grief reactions in adolescents included feelings of shock, sadness, guilt, injustice, anger, betrayal, struggles with “why” questions, and worrying about the impact of the death on others. For most young participants, the death was incomprehensible.

*“When you lose someone through suicide and when you’re a kid, you don’t really know what’s going on, you are so confused. Whether you’re nine or 17, death by suicide is, I think, the most confusing way of dying. Because you’re just left with so many questions and so many whys and what if and how. How could you be feeling like this and all this sort of stuff”*.

(Bea, A)

Despite many agonizing questions, a few young participants were more accepting of the death, as they could empathize with their deceased relative and perceived that they had no intention of hurting them. Still, for most young people the bereavement by suicide or other traumatic death was a new and radical experience, for which they had not yet developed a language, as highlighted by this mother: *“My daughter couldn’t say how she was feeling. All she could say was, ‘I have feelings’”* (Celeste, P).

Moreover, participants felt that the death had changed the young person’s identity. For example, those who had lost a parent had suddenly become half-orphans, and those who had lost a sibling were unsure if they were still a brother or sister. Overall, participants who had experienced more than one death reported that experiencing the death of a parent or sibling had a more severe impact than other deaths as the parent or sibling was a confidant or role model for values and norms. Some participants stated that experiencing a death by suicide differed from experiencing a death from natural causes.

*“[When] my grandfather passed away through cancer, there weren’t any of those questions. I was just a normal kid grieving a death, but when you’re grieving a death through suicide it’s like your brain is exploding all the time. You’re taken over by so many emotions, rather than just sadness or stuff like that”*.

(Bea, A)

One mother highlighted both the type and the emotional closeness of the relationship.

*“I think that the relationship to [the person] who died is very important with teenagers, and the depth that they feel and how close they were to that person”*.

(Fabienne, P)

Many young and parent participants emphasized that the bereavement compounded age-related challenges.

*“On top of that there’s obviously school and friendships and relationships and problems with my parents and all that sort of stuff. So, I think that came along when I was more of an older teenager, like 15 and now I’m 16”*.

(Bea, A)

*“When you are 15 year old, your brain is not developed until your twenties. I mean it’s hard enough being a teenager and getting through that and having to deal with something like that”*.

(Diane, P)

All young participants experienced long-term negative and/or positive effects (i.e., personal growth) from the death. Regarding negative effects, many participants particularly highlighted mental health issues such as struggles with post-traumatic stress, depression, anxiety, or self-harm.

*“She’s a very high functioning depressed young lady. She does what she has to do, and then she comes home, walks in the door and goes to bed. Then she sort of started binge eating and stuff like that. So, yeah, the poor little thing, she’s quite mixed up and messed up”*.

(Helene, P)

In addition, most young participants experienced strong long-term negative effects due to triggers, which could occur even several years after the death. They emphasized that “it never really goes away”. Triggers could be little or random things, related or unrelated to the death or the deceased person (e.g., a song on the radio) that ambushed the bereaved young person. Additionally, foreseeable events like anniversaries or holidays (e.g., Christmas) could trigger a return to the turmoil of their grief for young participants. Overall, while most participants acknowledged that the grief related distress had decreased over time, several participants stated that the impact of the triggers equaled or exceeded the intensity of the acute grief. As one participant said: *“When I do get upset, it hits me like a tsunami”* (Lucia, A). Young participants became to realize that the cycle of ongoing ups and downs was “*just going to be like that forever”* (Maggy, A).

*“There are times that you can recognize it, but then there are also other times where you just won’t expect it. Like, I was teaching the other day and a parent walked past who looked exactly like my dad and I was just like, what the fuck’s going on? It was back downhill again”*.

(Andy, A)

As most people in their social environment were not aware of the triggers, many young participants faced a dilemma about disclosing their triggers to others. Being open about them would allow others to be more sensitive and respectful towards the bereaved young person, which they would appreciate. However, this would require them to be open towards people whom they were unsure whether they could trust them. It would also provide opportunities for others to inquire about the bereavement, which they preferred to avoid. Nevertheless, those who had shared their triggers with friends reported being happy with it.

Most young and parent participants recognized lasting positive effects of the bereavement in young people, mostly in the context of personal growth. Participants experienced various aspects of personal growth, such as increased personal strength (including perception of self and finding new possibilities), interpersonal relationships (including increased compassion, altruism, and giving back to others), and a stronger appreciation of life. However, a few young participants and parents also expressed a concern about being too empathic towards others but not towards themselves.

*“I think you’re more acutely aware and in touch with your own emotions and other people than people who haven’t really been through it so young; you understand a lot more”*.

(Naomi, A)

*“My daughter has always been empathetic, but it has actually made her have better eye line on people and why they might behave in a particular way. I suppose my fear is that she doesn’t feel she has to be a saviour of other people and I’m fearful of that”*.

(Gaby, P)

To summarize this theme, the death of a close person was a new experience in the life of the adolescents, and it had an immediate and profound impact on their lives. The type of relationship and/or the cause of death were important issues in their grief experiences. The grief compounded identity–developmental challenges and led to long-term effects, both negative (e.g., response to triggers) and positive (e.g., personal growth).

### 3.2. Theme 2: The Death Differentiates You from Your Peers

Most young people emphasized that the death differentiated them from their peers in two ways. First, they felt that they were the only ones in their social environment who had lost someone they were close to. Second, they stated that other people treated them differently, compared to before the loss. Consequently, they felt lonely, disconnected or standing out. In some young participants, this aroused feelings of shame or fear of being judged by others, which in turn contributed to their social withdrawal.

*“I felt very alone in it. Because I’d never really heard of anyone losing a parent so young. It never happened to any of my friends or any of my family or anything”*.

(Lucia, A)

*“My daughter was in a basketball team that she’d been in for a few years. She just couldn’t go back. She didn’t want to face that group of girls, because they knew”*.

(Odette, P)

The young participants expressed a strong desire to be treated the same way they were before the death. They believed that this would help them to maintain a sense of normality and to find distraction from the grief. Many parents corroborated this need for normality, and a need in the young person to “escape” from being surrounded by the grief and/or being treated differently.

*“Yeah. It was really hard, because I just wanted to be able to get away from everything and just go to school and-but they just all treated me different. So I just couldn’t really go, because it was all about them thinking there’s something wrong and trying to fix it when I just wanted to go to school”*.

(Pamela, A)

*“He wanted some sort of normality and going back to school and doing those things was what he needed. His school friends were his family also and to be away from the sadness and the grief [at home] was to go to school and to be a normal kid. I think he wanted to switch off from it”*.

(Petra, P)

Further, young participants emphasized that the bereavement had a substantial impact on their friendships with peers. Participants felt little understood by their friends (“they don’t get it”) and in order to avoid unhelpful reactions, several participants started to conceal their feelings, which was also observed by parents.

*“At the weekends with friends, they’d be like, are you okay? I’d be like, yeah, I’m fine, but you would just use the partying as—yeah, I’m all good, I’m having a great time, but really inside you were dead”*.

(Naomi, A)

A few young participants reacted more distinctively to their loss, and/or anticipated being treated differently, by engaging in morbid jokes or artwork about death and suicide. This partly allowed them to express some of their feelings, and partly served as a test to see whom they could relate with about their grief. Some young people engaged in helping other bereaved young people even if that precluded finding help themselves, as emphasized by this participant.

*“And the boys, so we were just focusing on them, making sure they’re safe and okay. But at the same time we weren’t helping ourselves until now, there’s nearly two years and we’ve only just started to grieve”*.

(Jessy, A)

Many young participants narrowed down their circle of friends or social relationships. They reported that they could not deal with the stress they perceived from others, or lost interest in interacting with others, particularly those who could not understand or cope with their grief.

*“Friends that seem depressed, I will stop being friends with them because I’m too scared that they’ll leave, just like mum did”*.

(Rachel, A)

*“I lost a few friends. They approached me at one point and they were like, ‘these past few months, we never knew how you were going to be each day. Like, one day you would just act out and you would have a really short temper, and the next day you’d be really nice and we get along with you, and we just can’t deal with it anymore’. I was like, too bad, because it’s not really my fault. If you don’t want to deal with it, then we’re not going to be friends”*.

(Tania, A)

For some of the young participants, the bereavement experience was so overwhelming that they shut down or withdrew completely, including by seeking physical isolation or moving away from home. As one mother stated about her daughter: “*She just wanted to be herself, not the girl who’d lost her brother*” (Sheila, P). For some young participants, this met a need for working through their emotions, and the tensions felt at home, on their own.

*“When I went away, I realized how to be by myself, how to get myself through things, how to connect with people and build my family, not get in trouble with things and all that”*.

(Lucia, A)

*“He was old enough in some ways too, to make some decisions for himself and he obviously chose quite wisely. He just couldn’t take any more grief after a while and you can’t, I don’t think, as a child to be surrounded by that grief of parents and their friends and other people and everybody who comes to the house, or is around the house”*.

(Petra, P)

In summary, many bereaved young participants felt lonely and expressed a great need to be treated the same as they were before the death. Many young participants narrowed down their social relationships, or withdrew completely, because they felt poorly understood and/or feared being judged by others.

### 3.3. Theme 3: The Death Impacts on the Family System

Both the young participants and the parents underlined the impact of the death on the family system and discussed how this in turn further affected the bereaved adolescent. Above all, participants identified the loss of the family equilibrium as a crucial impact of the death. Participants highlighted that the bereavement changed the family functioning and dynamics in relationships. Participants came to realize that they had to rebuild their relationships as a family.

*“You’ve changed fundamentally in every single way and you have to learn who you are. You have to learn what you like, what you don’t like, your own limits and tolerances. At the same time, you’re trying to learn about this other person [i.e., bereaved adolescent]. They look and sound and seem to be the same person that they were before, but they’re not, because they’ve experienced the same loss as you in a different way. It’s changed them too. So, you’re trying to learn about you, this new you, because now you’ve got a new normal”*.

(Celeste, P)

Participants emphasized the occurrence of reciprocal worries between the bereaved young people and their immediate family members (mostly parents), and reciprocal struggles on how to support each other, or at least to spare others’ feelings. The bereaved young person would try to spare others, especially their parent(s) by being silent about their own grief (to one or both parents). Some young people stated that they believed that their parents would have been less impacted if they had died instead of, for example, their sibling. While some bereaved young participants felt betrayed by the deceased person and blamed a parent for the death, others strongly believed that they should not upset their parents, and/or should care for, or take responsibility for parents or siblings.

*“When they first die, it’s like the parents are a mess. They’re like-the teenager is expected to try and be strong for everybody and help plan everything”*.

(Pamela, A)

*“My son was worried about me, and he didn’t want me to have to look after him, when he knew that I was flat out. He’s a very sensitive, beautiful boy. I worry about him like nothing else now, but I can’t be paranoid”*.

(Kristie, P)

Participants noted that there were individual differences between family members regarding how to cope with their grief and the need and timing to talk about it.

*“One of the most challenging things I had was that each of my kids was different and needed different things. What was right for one—one of us needed the four of us to be together—was really bad for the older one, who was needing to get away and be separate. So it was like, how do I manage this—these competing needs?”*.

(Vanda, P)

The death had further consequences for parents as they also had to deal with other family members, practical arrangements (e.g., regarding the funeral), financial impacts and police inquiries. Overall, parents struggled with their own grief and/or mental health to the extent that they doubted their capacity and skills to support their bereaved children. In some parents this was compounded by other people providing “gratuitous” advice, or family members and/or the bereaved children blaming the parent for the death.

Conversely, some bereaved young people felt overlooked by their parents or family. Some also experienced a lack of privacy due to relatives or other visitors coming to their house, or relatives posting public messages on social media.

*“My mother was dealing with her[self], with me, with my older brother, so it was more of a ‘she needed to find herself’ before she could help us. She did the best for me so I wouldn’t end up in hospital but everything else she didn’t really care about”*.

(Wendy, A)

*“If we can’t cope, how do we expect our children to cope when they don’t have the same skills that we do and the same life experience”*.

(Yvonne, P)

Particular concerns regarding the impact of the death occurred in the context of health risk behaviours. Many young participants had engaged in rebellious behaviours (e.g., standing up against parents) or health risk behaviours, including acting-out and aggressive behaviour (such as fighting), reckless driving, and self-harm. Alcohol and substance use was particularly common. While some participants used alcohol or other drugs to numb the pain, others used them to conceal their feelings, to act “normal” and/or to find distractions from their grief. While young participants were aware of potential risks associated with their behaviour, some also minimized the risks or perceived these as less applicable to them.

*“I was definitely angry a lot. I needed some sort of outlet for that, but I didn’t—so I punched here and there, but nothing out of the ordinary”*.

(Peter, A)

Subsequently, many parents worried about the wellbeing and safety of their children, and started to doubt their parenting skills in terms of how to deal with the health risks in their children. Nonetheless, some parents understood the substance use of their children as a coping mechanism for their grief and/or as age-related behaviour. Some parents felt more at ease when they noticed that it was manageable, did not make their child’s situation deteriorate, and/or gave the child some benefits in terms of their behaviour or coping with their grief.

*“My daughter was smoking pot, she was stealing, she was smoking, she was drinking alcohol, she was 14 years old. She was stealing alcohol from my cabinet. I found it at the side of her water bottle and her bed. She was drinking that before she went to school. The absolute horror to me. I felt sick, you know. The pain again of what have I done wrong, as a parent?”*.

(Odette, P)

*“You’ve got to be really calm. Even though you want to scream at them because you find out they’re taking drugs, you’ve just got to wait until you can cope with that fact and how you’re going to talk about it so you don’t push them further in that direction”*.

(Zara, P)

Some parents negotiated with their children, trying to find a common ground (*“Self-destruction buttons aren’t going to work, I said, it’s to be a shared pain”,* Odette, P), and trod a fine line between providing advice and allowing the bereaved young people to make their own decisions, as highlighted by this parent: “*It’s that fine line, as a parent, how much you step in and how much you don’t”* (Fabienne, P). Both young people and parents highlighted that “*teenagers don’t always have the best relationships with their parents*” (Isabel, P), which hindered having open conversations about their bereavement.

*“They want to do something different to what mum’s doing. It’s part of the age and stage and sometimes it’s part of the personality too. It’s almost like, ‘oh I think this would be really good’, but if I suggest then it’s going to be a no”*.

(Vanda, P)

Some parents and young participants had engaged in constructive conversations between parent and child, about their deceased relative, feelings of guilt, blame and anger, and their relationship in the new family constellation. They felt that this had strengthened their bond, and perceived it as helpful in their grief as it helped to create a story and find meaning in the loss. Some parents noted that this process took several years as the young person’s notion of the death and the bereavement experience evolved as they grew older, as highlighted by this parent: “*Stuff has to keep being revisited as the years go on*” (Vera, P).

*“I feel like our family really banded together. I feel like that’s where I got the most support because we have a somewhat large family and I feel like at least we all understood what the others were going through”*.

(Amy, A)

*“She’s getting a picture of who he was because her memory—because I said to her, you’ve got a flawed memory of him because you knew him more and the more recent memory is the memory of a depressed man that was really, really struggling and you couldn’t see his generosity as much”*.

(Sabrina, P)

Overall, looking back several years after the death, some participants noted that they had moved forward, though “*you were not the people that you were before*” (Celeste, P).

*“I felt last year we turned the corner where it started to describe that it’s not dictating our life anymore. It’s influencing our life, but it’s not dictating. I actually feel, in a lot of ways, my children have lost five years of their lives, because they were so busy surviving this, where other people in their ages would have matured and had experiences. I actually feel all my children have lost time to shape themselves [as adults] because they were busy surviving what had happened to our family”.*

(Isabel, P)

To sum up this theme, the loss of the family equilibrium constituted the primary impact of the death. Young participants and parents commonly experienced worries and struggles regarding how to support each other, while acknowledging individual differences regarding how to cope with and the need to talk about their grief. Particular concerns were raised regarding health risk behaviour of bereaved adolescents. While parents struggled with their own grief and mental health, they also engaged with the bereaved children to find a new family balance.

## 4. Discussion

The study sought to broaden our understanding of bereavement by suicide or other traumatic deaths in adolescents by examining the views of adolescents and parents. The study findings clearly showed that the bereavement had a substantial impact on the lives of adolescents, adding to age-related issues, and beyond the intrapsychic grief experiences (e.g., feelings of sadness, guilt, anger) and coping styles, which are usually reported in the literature [7,20,21,44]. This study revealed that the death constituted a turning point in the adolescents’ lives, which differentiated them from their peers and ruptured the family equilibrium.

For the young participants, experiencing a suicide or other traumatic death was a novel life event for which they were not prepared; to the extent that many felt overwhelmed. Subsequently, they struggled to find a language to express themselves and ways to deal with it. Previous research had reported that the social circle of adolescents bereaved by suicide narrowed [22,45]. However, young participants in this study withdrew not only because of the perceived unavailability or unhelpfulness of their social network [45], or fears of abandonment [22], but also as a deliberate choice to have time and space to deal with their grief feelings on their own and to “escape” from the grief and stress experienced from others. While some parents in this study had experienced this as a source of concern, many young participants and parents reported that the withdrawal probably was appropriate at that time, and helped the bereaved adolescent find their way of coping with the loss. As such, this finding diverges from other suicide bereavement studies (albeit with adult populations, e.g., [46]) which emphasized the positive association between sharing grief experiences and grief outcomes. Our study suggests that a more nuanced perspective on the importance of sharing grief reactions in adolescents might be needed. The finding may be in line with results from our previous research, which found high levels of self-reliance in bereaved adolescents [26]. Further research may investigate the relationship between self-reliance and coping in adolescents bereaved by suicide and traumatic deaths.

Many young participants reported having engaged in health risk behaviours after the loss, especially the use of alcohol or other drugs, a finding that was confirmed by parents in this study and by previous studies [22,45,47]. While substance use and reckless behaviour can be age-related experimentation [48], many young participants and parents in this study reported it as unusual and excessive. Some young participants seemed to use it to numb the pain and/or as an acting out mechanism. Though the health risk behaviours seemed to abate over time as revealed in this study, other studies have highlighted the risk of long-term mental health problems in the context of substance use and other risk behaviours (such as getting involved in violence or inflicting injuries to others) after loss by suicide or other traumatic death in young people [49,50].

Several adolescents reported having engaged in artistic and/or morbid humoristic expressions of their grief, a topic rarely mentioned in the literature on adolescents’ grief after traumatic death. Though such grief expressions may not be limited to adolescents, they may be highly relevant in this age group as adolescents may experience the same grief feelings as adults; however, they may express them differently [18]. In this study, adolescents used art and humor to express their grief in a safe way and explore potentially supportive connections with other people. According to Cox [51], bereaved young people may be drawn to artistic and symbolic expressions, which they may experience as less threatening, and not dependent on their verbal fluency to talk about the loss. Similarly, humor can be a way of simultaneously expressing and hiding grief, and channeling uncertainties or anxieties [51]. Adolescents may also use humor to strengthen relationships and to ameliorate the effects of stress [52]. It allows them to address a topic that they feel uncomfortable with without being accountable for the content [52]. As such, artistic and humoristic expressions of grief, especially after suicide and traumatic deaths, may facilitate connecting with others and decrease the sense of loneliness, which was a prime feeling reported by young participants in this study.

In line with the literature [7], young participants in this study felt that the suicide or other forms of traumatic death of a sibling or parent had a stronger impact than other deaths. The importance of the sibling bond was evident in the study by bereaved siblings saying that if they had died, the parents would be less upset. For most adolescents, the sibling relationship is a constant, interactive and dyadic relationship, which is crucial in the process of identity formation and acquiring life skills [53,54]. It is often the longest interpersonal bond they have had [55], and as mentioned by young participants and parents in this study, “the sibling has always been there”. Hence, the death of a sibling affects identity formation as the bereaved sibling must redefine their roles without their primary referent [53]. Siblings bereaved by any type of death, but especially by suicide and other traumatic deaths, may struggle with mental health issues such as depression and anxiety above any pre-loss health conditions, either in a short- or long-term [55,56,57]. Grief and mental health outcomes in siblings may be affected by their self-concept and family functioning, though further research in this field is necessary [7,56,57].

Death of a parent was the most frequently experienced loss in this study, and there is ample evidence of the severity of the impact of parental loss during adolescence [7]. In line with the literature, adolescents in this study reported long-term risks of mental health problems in addition to short-term risks of anxiety, depression and substance abuse [11,58]. The literature also highlights the risk of decreased long-term developmental competencies, such as diminished educational and work aspirations, in the aftermath of parental suicide [58]. In addition, several young participants and parents referred to self-harming behaviour in bereaved adolescents to the extent that some parents feared that the bereaved adolescent child might die as well. According to the literature, adolescents bereaved by parental suicide have a two- to three-fold risk of suicide, attempted suicide, and psychiatric hospitalization, independent of history of familial mental health problems [50,59,60], with bereaved boys and girls having similar risks [61]. This finding points at the crucial need for specialist help for these adolescents.

Some young participants who had experienced different losses reported that in their experience a death by suicide was more impactful than other losses, which taps into a much-debated topic in the literature regarding the “uniqueness” of loss by suicide [15,62]. Based on findings from controlled quantitative studies, there is growing evidence that there are more commonalities than differences between different groups of bereaved people, based on cause of death, regarding major grief themes, grief processes and grief outcomes [8,9,63]. However, there is also evidence of increased risk of mental health complications and suicidal behaviour in adolescents bereaved by suicide or other external causes compared to natural deaths (e.g., [50,59,60]), a concern that was also strongly voiced in this study.

However, quantitative studies cannot grasp nuances of personal experiences, and personal accounts of individuals bereaved by suicide and narratives of clinicians seem to stress features that are experienced as unique or central to the loss by suicide in adults (e.g., [9,64]. Similarly, qualitative studies with adolescents have reported that they experience feelings such as guilt, shame, anger, rejection, and struggles with “why” questions, features that may be more pronounced, though not unique to suicide bereavement [10,22,45,65]. Still, to the best of our knowledge, qualitative comparisons between different forms of bereavement in adolescents (as well as in adults) are still to be conducted. Nonetheless, there is increasing evidence of the importance of the emotional closeness of the relationship with the person who has died as a factor contributing to the impact of the loss in adolescents, independent of kinship or cause of death, though further research is also needed here [66,67].

Although studies have looked at the long-term grief and mental health effects of loss by suicide in adolescents (e.g., [58]), participants in this study reported long-term negative experiences, especially through the effect of triggers, a finding that has received little attention in the adolescent bereavement literature. Building on the trauma literature, Cohen and Mannarino [68] distinguished between triggers that were related to the trauma of the death, the loss experience, and the subsequent changes in the lives of the bereaved adolescents. Given the debilitating effect of such triggers, the finding has clinical importance and warrants further investigation in this population.

In line with previous research (e.g., [10]), young participants in this study emphasized their experiences of personal growth stemming from life lessons learned from the loss, including increased appreciation of life and relationships, and overall increased maturity [69]. It has been suggested that the experience of personal growth may buffer the impact of grief, mental health and suicidal ideation in adolescents bereaved by suicide [10]. However, some young participants in this study noted that they had become more empathic towards other people, but not towards themselves. Overall, the relationship between cause of death and personal growth in adolescents is not clear [70,71]. In line with the literature on personal growth in adults bereaved by suicide, the scant literature on adolescents suggests that intermediate levels of grief-related impact (contrary to high or low levels) and actively engaging and struggling with the grief process may facilitate personal growth in adolescents [71,72,73]. Further research may elucidate which psychological (e.g., coping styles) or social factors (e.g., family relationships, help-seeking) may contribute to personal growth in bereaved adolescents, and how this may inform interventions for this population [71,72,73,74].

Many young participants reported feeling different from their peers, which constituted a crucial finding regarding the relational impact of the loss and has been found in a few other qualitative [23,75] and quantitative studies [76]. Importantly, the bereaved young participants felt alone and disconnected from their peers, a phenomenon known as “secondary losses” [77] (p. 200), which contributed to their social withdrawal. Parents participating in the study corroborated the adolescents’ needs to be treated the same as before the loss. In line with experiences of young participants in this study, the literature has argued that schools can be a supportive environment for bereaved young people, as a place where they can experience a sense of normality and routine, away from the grief at home [78,79]. Similarly, qualitative research has indicated that finding distractions from the grief is of equal importance for adolescents in coping with the loss as addressing the grief directly [10,80], which is in line with the oscillating grief dynamics described in the Dual Process Model of coping with bereavement [81].

Most young participants and parents emphasized the devastating impact of the suicide or other traumatic death on the family equilibrium, a finding little noted in the literature [21,82]. The young participants had lost their emotionally safe and trusted environment and tried to deal with and/or help their parents or siblings. Conversely, parent participants reported about their worries and challenges on how to parent their bereaved adolescent while looking after their own grief and mental health [82,83]. Parental grief and mental health problems additionally affect functioning in bereaved adolescents [13]. Importantly, the loss of the equilibrium created a limbo that was maintained by individual differences in grieving needs and tempos. Still, finding a new family balance was a common goal of adolescents and parents in this study. The importance of the finding is underscored by the fact that adolescents perceive their parents as the major source of help after any type of bereavement [84], and parents are a major facilitator of referring bereaved adolescents to professional help [26,27].

Many parents (i.e., mothers) in this study worried about the—from their perspective—apparent lack of grieving behaviour or the excess of it, in terms of health risk behaviours, in their adolescent children, and their own reduced emotional availability. This finding is in line with research revealing that mothers are likely to take up the caring role and responsibility for daily routine for their bereaved children [18], and research that has found that externalizing problems in bereaved siblings was associated with distress in mothers but not in fathers [85]. However, our finding also seems to contradict other research stating that spouses of parents who died by suicide exhibit few mental health problems and are likely in a position to buffer the impact of the loss in their bereaved children [86]. These contradictory findings may point at the complex and dynamic nature of the impact of a suicide on a family with adolescent children, a field greatly in need of further research.

### Limitations

The study recruited voluntary (mostly female) young participants and parents (all mothers), which may have introduced a selection bias. Those who were more verbally skilled may have been more likely to volunteer, contrary to those who were more distressed at the time of recruitment. The study relied on self-report of experiences up to 10 years ago, which is open to recall bias. Additionally, although participants had ample opportunity to talk about their experiences, it is possible that some important issues were not shared. Nonetheless, the study recruited a purposive sample from across the country using multiple recruitment channels and interview settings to ensure variation in the sample and resulting in a rich data set. However, despite our efforts to recruit mothers and fathers, only mothers volunteered. As there might be differences in views held by mothers and fathers [87,88], future studies should include fathers. Further, the sample consisted of participants mainly bereaved by suicide, and some participants bereaved by other traumatic deaths. Although grief reactions after suicide and other traumatic deaths are mostly similar [15], the study design did not allow examining this. Further studies may also assess the views held by adolescents vs. those of parents.

## 5. Conclusions

The study clearly demonstrated the devastating impact of the death on the adolescents to the extent that it turned around their lives, differentiated them from their peers and ruptured the family equilibrium. Importantly, the adolescents tended to take charge of their own situation: they were active agents rather than passive recipients of grief [89]. They reacted to a death, which they could not comprehend, in a way that they felt was best at that time, even if that involved reactions such as withdrawal or substance use, which were worrisome from a parents’ perspective. Hence, in order to help bereaved adolescents and to improve their coping mechanisms, it is of utmost importance to understand the impact of the death from within their agency and experience.

By examining the views of adolescents and parents alike, the study demonstrated that the adolescents’ grief and coping was intertwined with their close relationships. Hence, their grief must be studied within the context of their immediate social environment, which affects the development of their personal identity, social independence and maturity. The study findings add to the scant literature in this field [26,30,90], indicating that support for adolescents bereaved by suicide or other traumatic deaths should look beyond individual coping and incorporate the quality of the relationships between the adolescent and parent/family, for example via family programs or parenting support. Given the limited knowledge of what constitutes effective interventions in this field [91], research regarding the development and evaluation of interventions along these lines is urgently needed.

## Figures and Tables

**Table 1:** Description of themes.

Theme	Content
**1. The death is a life-changing experience**	- The death turns life upside-down - The importance of the type of relationship and cause of death of the deceased person - The grief compounds age-related challenges - The grief has long-term negative and positive impacts
**2. The death differentiates you from your peers**	- Feeling alone - Wanting to be treated the same as before - Impact on friendships
**3. The death impacts on the family system**	- Loss of the family equilibrium, and worries and struggles to support each other - Consequences of bereavement for parents and their ability to support their children - Concerns regarding health risk behaviours in bereaved young people - Parents and bereaved young people engaging to find a new balance

## Supplementary Materials

The following are available online at https://www.mdpi.com/1660-4601/17/24/9356/s1, Table S1: Sociodemographic characteristics of participants (*n* = 38).
