# Supplementary material for: “It Changes Your Orbit”: The Impact of Suicide and Traumatic Death on Adolescents as Experienced by Adolescents and Parents
Source: Int J Environ Res Public Health. 2020 Dec 14;17(24):9356. doi: 10.3390/ijerph17249356 (PMC7765017; doi:10.3390/ijerph17249356)
Supplement: Supplementary file 1 [file ijerph-17-09356-s001.pdf]

Supplementary Table S1: Sociodemographic characteristics of participants (N = 38)

	**Adolescents (n = 20)**	**Parents (n = 18)**
**Age (years)**	Range 14-26; M=19.50 (SD=2.95)	Range 43-60; M=53.20 (SD=4.35)
**Gender**	F/M: 16/4	F/M: 18/0
**Years since death**	M=3.92 (SD=2.49)	M=5.31 (SD=2.89)
**Cause of death of close person**	Suicide (n = 18)<br>Accident (n = 2)	Suicide (n = 13)<br>Accident (n = 2)<br>Manslaughter (n = 1)<br>Illness (n = 1)<br>Undetermined (n = 1)
**Relationship of the bereaved adolescent with deceased person**	Father (n = 9)<br>Brother (n = 2)<br>Sister (n = 2)<br>Mother (n = 2)<br>Other family (n = 2)<br>Friend (n =2)	Father (n = 10)<br>Brother (n = 4)<br>Sister (n = 4)
